# The laryngectomee guide for COVID-19 is now available in Portuguese

**DOI:** 10.1016/j.bjorl.2021.01.003

**Published:** 2021-02-10

**Authors:** Itzhak Brook, José Guilherme Vartanian, Rui Imamura

**Affiliations:** a Georgetown University School of Medicine, Department of Pediatrics, Washington, United States; b Centro Oncológico A. C. Camargo, Departamento de Cirurgia de Cabeça e Pescoço e Otorrinolaringologia, São Paulo, SP, Brazil; c Universidade de São Paulo, Faculdade de Medicina, Hospital das Clínicas, Departamento de Otorrinolaringologia, São Paulo, SP, Brazil

Dear editor,

In a recent editorial in BJORL, Chone^1^ commented on the increased mortality from head and neck cancer due to the COVID-19 pandemic. Not only head and neck cancer patients who are awaiting oncological treatment, but also those already operated need support and orientation during this pandemic.

In this context, it is a pleasure to announce that “The Laryngectomee guide for COVID-19” from Dr. Ithzak Brook is now available in Portuguese. The translation into European and Brazilian Portuguese was supervised by Drs. Elizabeth de Oliveira Costa, Gabriela Torrejano, Pedro Pestana, Jaqueline Carmona, José Guilherme Vartanian and Rui Imamura.

It is available (FREE) at: https://bit.ly/3eFcYPN. A paperback edition can be obtained at https://bit.ly/3pklpVJ.

This open access e-book provides valuable information on how laryngectomized and tracheostomized people should deal with the COVID-19 pandemic. With it, patients will receive tips on how to prevent infection by SARS-CoV-2, the proper use of masks and tracheostoma protectors, in addition to other important issues, including: tracheostoma care, how to deal with depression and social isolation during the pandemic, care when leaving home and guidance in case of need for hospitalization, among others.

We believe this guide will be helpful for both physicians and patients.

## Conflicts of interest

The authors declare no conflicts of interest.
